# NEMO score in nailfold videocapillaroscopy is a good tool to assess both steady state levels and overtime changes of disease activity in patients with systemic sclerosis: a comparison with the proposed composite indices for this disease status entity

**DOI:** 10.1186/s13075-019-2032-6

**Published:** 2019-11-29

**Authors:** Francesca Pignataro, Wanda Maglione, Antonina Minniti, Domenico Sambataro, Gianluca Sambataro, Francesco Campanaro, Gabriele Valentini, Claudio Vitali, Nicoletta Del Papa

**Affiliations:** 1 Department of Rheumatology, UOC Day Hospital of Rheumatology, ASST G. Pini-CTO, Milan, Italy; 2 Department of Clinical and Experimental Medicine, Internal Medicine Unit, Section of Rheumatology, University of Catania, Catania, Italy; 3 Department of Clinical and Experimental Medicine, Regional Referral Center for Rare Lung Disease, University of Catania, Catania, Italy; 4 Department of Internal Medicine, Rheumatology Unit, 2nd University of Naples, Naples, Italy; 5 ‘Mater Domini’ Humanitas Hospital, Rheumatology Outpatient Clinics, Castellanza, Italy

**Keywords:** Nailfold videocapillaroscopy, Microhaemorrhages, Microthromboses, Disease activity, Systemic sclerosis

## Abstract

**Background:**

In previous studies, we demonstrated that the NEMO score, i.e. the cumulative number of microhaemorrhages (MHEs) and microthromboses (MTs), observed in nailfold videocapillaroscopy was a good indicator of the steady state level of disease activity (DA) in patients with systemic sclerosis (SSc) when the European Scleroderma Study Group (EScSG) index was considered the gold standard.

**Aim of the study:**

To verify whether the NEMO score could be (i) a valid tool to assess DA, even when the modified European Scleroderma Trials and Research (EUSTAR) index was considered to be the comparator, and (ii) a sensitive method to capture the DA overtime changes.

**Patients and methods:**

The NEMO score and the EScSG and EUSTAR indices were contemporarily assessed at baseline (T0) and after a follow-up of 4–56 months (T1) in 98 patients with SSc. The differences (Δ) between the T1 and T0 values of the NEMO score and the EScSG and EUSTAR indices were calculated and compared to each other.

**Results:**

NEMO score values were very closely correlated with the corresponding values of the EScSG and EUSTAR indices both at T0 and T1 observations (*p* < 0.0001 in all cases with the exception of the correlation with EScSG values at T1 (*p* < 0.03)). The values of the two composite DA indices were also strictly related to each other in both T0 and T1 observations (*p* < 0.0001).

Receiver operating characteristic (ROC) curve analysis showed the NEMO score had a good sensitivity and specificity in classifying patients with a predefined level of DA (scores ≥ 3.0 and ≥ 2.5 for the EScSG and EUSTAR indices, respectively, *p* < 0.0001 in both cases).

Δ values of the NEMO score were significantly correlated with the corresponding values of both the EScSG and EUSTAR indices. Weighted Cohen’s *k* level of agreement between Δ values of the NEMO score and those of the EScSG and EUSTAR indices was moderate (0.55 and 0.59, respectively).

**Conclusions:**

NEMO score proves to be a feasible, non-invasive, and valid tool to assess steady state levels and changes over time of DA in patients with SSc. Thus, it can represent an alternative or complementary method to measure this disease status entity in this disorder.

## Introduction

Nailfold videocapillaroscopy (NVC) is a feasible method that allows the observation and follow-up of the microvascular changes that mark the course of systemic sclerosis (SSc) [1]. Some peculiar NVC abnormalities, namely dilated capillaries and avascular areas, are so specifically associated with the diagnosis of SSc that they have been included as a separate item in the 2013 classification criteria for this disorder [2]. Furthermore, different NVC aspects have been reported to be predictive of specific disease-related outcomes and manifestations [3, 4]. Finally, it has been suggested that specific NVC pictures may characterise different phases of the disease. Therefore, early, active, and late NVC patterns have been carefully defined. According to this classification, the active pattern is characterised by the presence of numerous ectasic and giant capillaries (GCs), microhaemorrhages (MHEs), microthromboses (MTs), and scattered avascular areas [5].

In a previous cross-sectional study [6], we demonstrated that among the different abnormalities that define the active pattern of NVC, the cumulative number of MHEs and MTs (the so-called NEMO score) was closely related with disease activity (DA), assessed by the European Scleroderma Study Group (EScSG) index, which was considered at that time to be the reference composite scale to measure this disease status entity [7, 8]. In a subsequent study, the NEMO score was validated as a steady state measure of DA, again using the EScSG index as the comparator, in two separate cohorts of patients, the first one prospectively collected in the centre where the first study was carried out, and the second one retrospectively analysed in a different centre [9].

Although the EScSG index has been used to assess DA in SSc in many studies, its validity has been criticised. A new modified index derived from the analysis of a cohort of paper patients selected from the European Scleroderma Trials and Research (EUSTAR) database was then proposed and validated (EUSTAR activity index) [10].

Since the new index is somewhat different from the previous EScSG index, in the present study, we decided to test whether the NEMO score could be a valid tool for the steady state assessment of DA when the new index is considered the gold standard comparator. An additional aim of the present study is also to assess the validity of the NEMO score as a transition index, i.e. its ability to measure overtime changes of DA, even in comparison with the contemporary changes of both the EScSG and EUSTAR indices.

## Patients and methods

### Patients

The cohort of patients of this study is the same as the one used in the validation study of the NEMO score [9]. This cohort was initially composed of 102 prospectively collected patients with SSc who were referred to the Scleroderma Clinics of the Rheumatic Disease Unit of the Gaetano Pini Institute of Milan. All enrolled patients met the American College of Rheumatology/EUropean League Against Rheumatism (ACR/EULAR) classification criteria for SSc [2], and they were also sub-classified as having limited cutaneous SSc (lcSSc) or diffuse cutaneous SSc (dcSSc) according to the LeRoy et al. criteria [11]. At the time of the enrolment, it was preliminarily established that around half the patients with inactive disease (EScSG score < 3) and a similar proportion of patients with active disease (EScSG score ≥ 3) would be included [7, 8].

Exclusion criteria were the concomitant conditions that may potentially cause additional microvascular changes, such as diabetes, smoking and onychophagic habitus, presence of anti-phospholipid antibodies, and pregnancy [12–15]. Current treatment with beta-blockers was also an exclusion criterion because it is well known that this drug may cause or exacerbate Raynaud’s phenomenon (RP) [16].

At the time of study enrolment, 37 of the 102 patients were receiving treatment with infusions of intravenous prostanoids (32 with monthly iloprost and 5 with weekly alprostadil), and 8 were taking bosentan. It was decided for ethical reasons to continue these vasoactive treatments during the study. Furthermore, all of the patients were receiving stable treatment with low-dose acetylsalicylic acid and calcium channel blockers.

After the first observation time (T0), a second observation time (T1) was planned and completed after 4–56 months in 98 patients of the initial cohort. Three patients dropped out of the follow-up for unknown reasons, and naturally, the data from these three patients collected at T0 were excluded from the final analysis.

### Clinical work-up and assessment of disease activity

The EScSG and EUSTAR indices were taken as the reference tools (gold standard) for DA assessment both at T0 and T1 clinical observations. These are composite scoring systems that include a number of clinical, laboratory, and instrument items [7, 8, 10]. According to the authors’ indication, scores ≥ 3.0 and ≥ 2.5 allow capturing patients with a significant level of DA when the EScSG and EUSTAR indices are respectively applied [10, 17].

At T1, the NEMO score and EScSG index were assessed together with the new EUSTAR index. This latter index was also retrospectively assessed in the same patients at T0, while the EScSG scale had been measured at the enrolment.

NVC examination was done in the same month in which the clinical data needed for the EScSG and EUSTAR scores definition were collected.

The modified Rodnan skin score (mRSS), which is included among the items of both DA indices, was obtained by experienced physicians (WM, NDP). It is well known that the mRSS is a valid and reliable method of assessing skin involvement in SSc, but its precise evaluation is a difficult challenge in unexperienced hands [18].

### Nailfold videocapillaroscopy

**Fig. 1:**
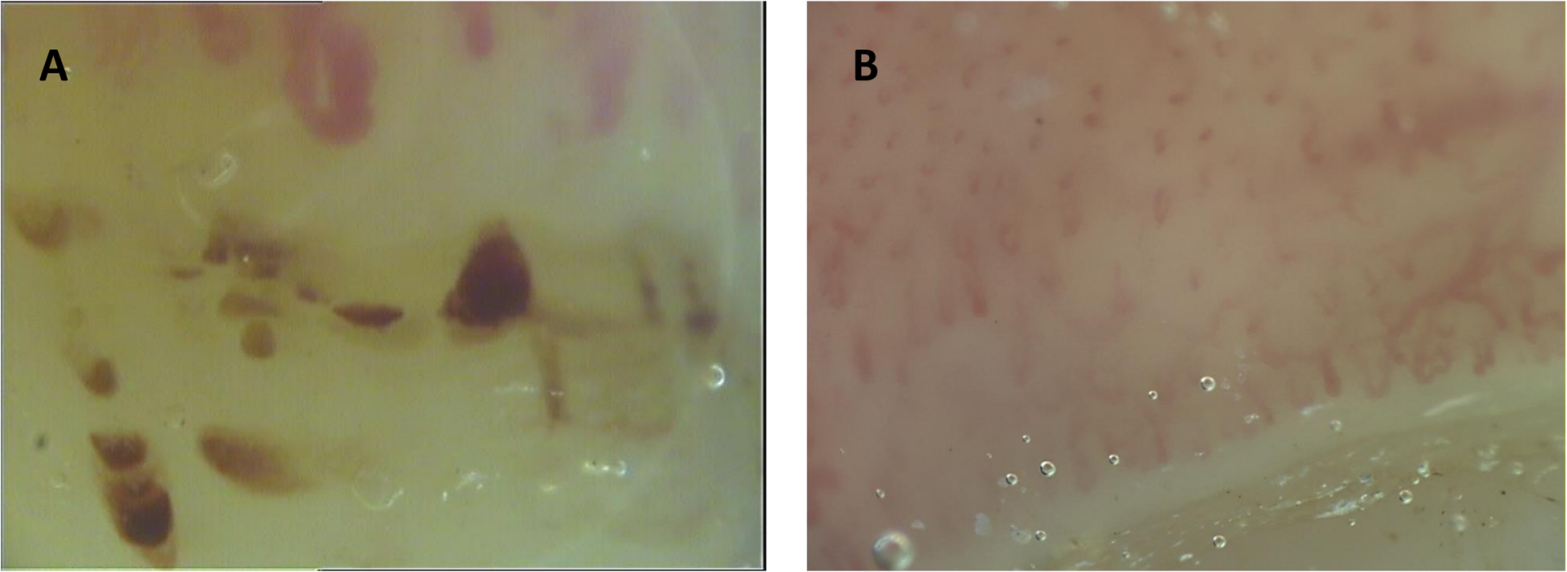
Example of NVC images showing a high NEMO score (**a**) and a low NEMO score (**b**). Numerous synchronous MHEs are observed aligned in the distal row of capillaries at the same level (panel **a**). Presence of altered architecture of capillaries in absence of visible MHEs and MTs are observed in panel **b**. NVC, nailfold videocapillaroscopy; NEMO, number of microhaemorrhages; MHEs, microhaemorrhages; MTs, microthromboses

A videocapillaroscope with a × 200 magnification lens was used to examine the nailfold capillary bed of all fingers of both hands, excluding thumbs, of each patient. Each digit was positioned in such a way that the capillaroscopic light was 90 degrees incident on the centre of the nailfold. Four adjoining 1-mm fields—two on the right and two on the left side, for a total extension of 4 mm starting from the middle of the nailfold—were examined. The derived digital images were stored and analysed using dedicated software (VideoCap; Scalar Co. Ltd., Tokyo, Japan). Only one experienced investigator (FP) was responsible for reviewing the stored NVC images of all the patients included in the study. For each patient, the NEMO score was calculated by counting the total number of MHEs and MTs observed in the distal row of capillaries aligned at the same level in the images obtained from the eight fingers of both hands. A score of 1 was given to each separate MHE and MT, independently of its size. In Fig. 1, typical examples of patients with high and low NEMO score (panels a and b, respectively) are shown.

### Statistical methods

Statistical analysis was performed according to standard procedures using MedCalc software package, 2014 version (MedCalc® Inc., Ostend, Belgium). Spearman’s rank correlation was used to assess the relationship between NEMO scores and either EScSG or EUSTAR scores at T0 and T1 observation. The use of this non-parametric correlation method was adopted because NEMO score variable did not have a normal distribution (Shapiro-Wilk test, *p* > 0.05).

Receiver operating characteristic (ROC) curves were constructed, and the areas under the curves (AUCs) were calculated by plotting the sensitivity and 1-specificity values of the NEMO score in correctly classifying patients with pre-defined levels of DA (scores ≥ 3.0 and ≥ 2.5 for the EScSG and EUSTAR indices, respectively) [10, 17]. The Hanley-McNeil test was applied to verify the presence of significant differences between the AUCs obtained from the analysis.

The differences (Δ) between the T0 and T1 values of the NEMO score and the EScSG and EUSTAR indices were calculated. The overtime variations of EScSG and EUSTAR indices were considered as standard measures of DA variations. These Δ values (T1 minus T0 values) of both composite activity indices were compared with the corresponding values of the NEMO score, to verify whether this NVC parameter maintains its validity to assess DA in SSc also as a transition index. Although Δ values obtained from the comparison from the T1 and T0 values of the NEMO score and the EScSG and EUSTAR indices were discrete variables, their distributions appeared to be normal (Shapiro-Wilk test, *p* < 0.001 for the NEMO score and *p* < 0.02 for both the EScSG and EUSTAR indices). As a consequence of this result, we applied linear regression analysis for the comparisons between the Δ values of these variables.

Δ values of both composite activity indices were also tested for their levels of agreement with Δ values of the NEMO score by using weighted Cohen’s kappa (*k*), after a subdivision of all the three parameters in quartiles. Cohen’s kappa levels were conventionally considered as indicative of good agreement between 0.61 and 0.80 and of moderate agreement between 0.41 and 0.60.

No correction of the statistical results was made for the presence of missing values, because there were no missing data in our database. Bonferroni’s correction for multiple comparisons was also applied when indicated.

### Ethical rules

This study was conducted according to the Helsinki Declaration and approved by the ethics committee of the ‘Azienda Socio Sanitaria Territoriale Lombardia, Centro Specialistico Ortopedico Traumatologico Gaetano Pini’ of Milan, where the study was carried out and where all the study patients were recruited. Written informed consent was obtained from all of the enrolled patients.

## Results

The main demographic and clinical characteristics of the patient cohort recorded at the enrolment time are reported in Table 1. The prevalence in the cohort of the different items included in both the EScSG and EUSTAR composite indices—again at T0 clinical observation—is also shown in Table 2.

**Table 1:** Demographic and clinical characteristics of the cohort of patients with SSC enrolled in the study

Numbers of patients	98
Male/female ratio	8/90
Median age, years (range)	58 (21–84)
Median disease duration, years (range)	6 (0–26)
Median T1-T0 interval, months (range)	12 (4–56)
lcSSc/dcSSc	48/50
Autoantibodies	
ACA, *n* (%)	42 (42.8)
Anti-Scl-70, *n* (%)	50 (51)
Others, *n* (%)	6 (6.1)
NVC patterns	
Early, *n* (%)	16 (16.2)
Active, *n* (%)	42 (42.8)
Late, *n* (%)	40 (41)
Patients on prostanoid therapy, *n* (%)	32 (32.6)

*dcSSc* diffuse cutaneous systemic sclerosis, *lcSSc* limited cutaneous systemic sclerosis, *ACA* anti-centromere antibody, *ANA* antinuclear antibodies, *NVC* nailfold videocapillaroscopy

**Table 2:** Prevalence in the cohort of the different items included in DA composite indices at T0

	Item	Number (%) of patients
In EUSTAR index only	mRSS > 18	7 (7.1)
	mRSS up to 18	91 (92.9)
	Tendon friction rubs	29 (29.6)
	CRP > 1 mg/dL	12 (12.9)
	DLCO < 70% of the predicted value	47 (48)
In EScSG index only	mRSS > 14	12 (12.9)
	Scleredema	60 (61.2)
	∆ Vascular	52 (53.0)
	Arthritis	23 (23.5)
	DLCO < 80% of the predicted value	67 (68.4)
	∆ Cardiopulmonary	18 (18.4)
	ESR > 30 mm/1st h	25 (25.5)
	Hypocomplementaemia (C_3_ and/or C_4_)	11 (11.2)
In both EScSG and EUSTAR	∆ Skin	27 (27.6)
	Digital ulcers	30 (30.6)

*EUSTAR* European Scleroderma Trials and Research, *EScSG* European Scleroderma Study Group, *mRSS* modified Rodnan skin score, *CRP* C-reactive protein, *ESR* erythrocyte sedimentation rate, *DLCO* diffusing lung capacity for carbon monoxide; ‘∆,’ difference of the parameters between two consecutive observations

At T0 observation, the NEMO score showed a highly significant correlation with both the EScSG and EUSTAR indices. At T1, the NEMO score maintained the same highly significant level of correlation with the EUSTAR index, while the correlation is less strong with the EScSG index. The two composite DA indices were also significantly correlated to each other in both observation times (see Table 3).

**Table 3:** Correlation coefficient (Spearman’s *R*) and its statistical significance (*p*) between values of the two composite DA indices and those of the NEMO score at T0 and T1 observations

	T0		T1	
	NEMO score	EScSG index	NEMO score	EScSG index
EScSG index	0.67 (*p* < 0.0001)		0.25 (*p* < 0.03)	
EUSTAR activity index	0.62 (*p* < 0.0001)	0.69 (*p* < 0.0001)	0.47 (*p* < 0.0001)	0.45 (*p* < 0.0001)

*DA* disease activity, *EScSG* European Scleroderma Study Group, *EUSTAR* European Scleroderma Trials and Research, *NEMO* cumulative number of microhaemorrhages and microthrombosis

**Fig. 2:**
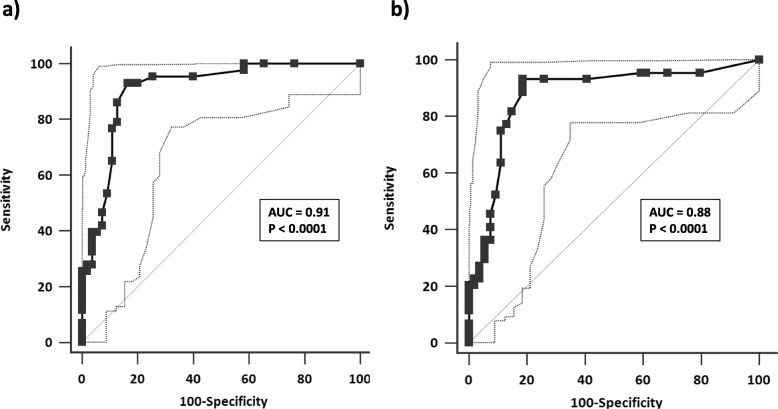
ROC curves obtained by plotting sensitivity and 1-specificity of NEMO score in correctly classifying SSc patients with predefined levels of DA, i.e. EScSG index ≥ 3 (**a**) and EUSTAR index ≥ 2.5 (**b**). AUC, area under the curve. Dotted lines represent 95% confidence interval of AUC

The ROC curves derived by plotting sensitivity and 1-specificity of NEMO score in correctly classifying active patients, i.e. patients with values ≥ 3.0 and ≥ 2.5 obtained by applying the EScSG and EUSTAR indices, respectively, are shown in Fig. 2. There is no significant difference between the two AUCs. NEMO scores ≥ 8 have a good sensitivity-specificity balance for this purpose (sensitivity 93.0% and 90.9%, specificity 81.8% and 81.5% in classifying patients with an EScSG index ≥ 3.0 and a EUSTAR index ≥ 2.5, respectively).

**Fig. 3:**
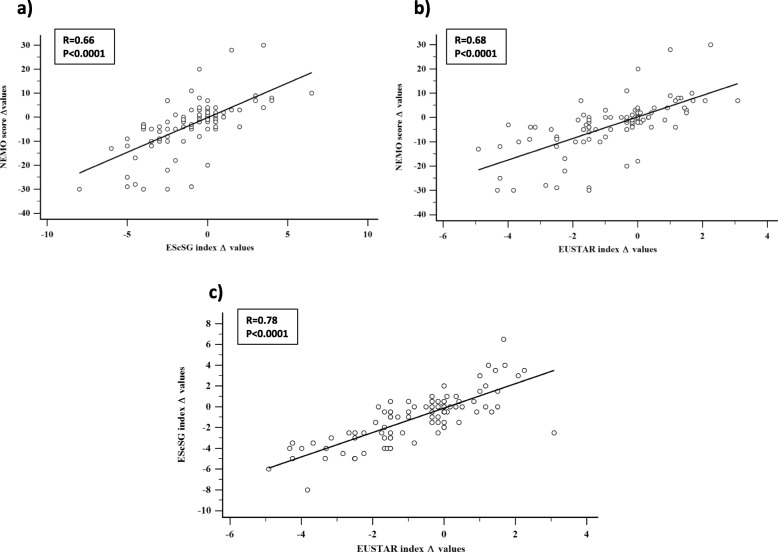
Linear regression analysis obtained by plotting Δ values (T1 minus T0 values) of the NEMO score and the corresponding values of the EScSG and EUSTAR DA indices (**a** and **b**, respectively). The same linear regression plot has also been obtained from Δ values of the EScSG and EUSTAR indices (**c**)

The linear regression analysis between Δ values of the NEMO score and the corresponding values of the EScSG and EUSTAR indices showed a highly significant level of correlation with both these DA composite tools. Even the two DA indices were similarly correlated to each other (Fig. 3).

The weighted Cohen’s *k* level of agreement between the NEMO score and the two DA indices, after the subdivision in quartiles of all the three parameters, was moderate, but close to the limit of good agreement [*k* 0.59 (95% CI 0.48–0.70) and 0.55 (95% CI 0.44–0.66) with the EUSTAR and EScSG indices, respectively]. Notably, a comparable level of *k* agreement was also observed between the two composite DA indices [*k* 0.60 (95% CI 0.49–0.71)].

The quartile subdivision also made it possible to define range values of the analysed parameters which might be somewhat indicative of stable levels or changes of DA. Additional file 1: Table S1 shows all the range values that define the quartiles for the NEMO scores and the EScSG and EUSTAR indices.

## Discussion

The definition of DA in systemic autoimmune diseases (SADs) is actually a difficult challenge. Since specific clinical or biological markers able to assess the degree of overall systemic activity in SADs do not exist, the measurement of this disease status entity can be obtained by developing specific criteria for each disorder, and this has been done for most of the SADs, including rheumatoid arthritis [19], systemic lupus erythematosus [20], and Sjögren’s syndrome [21]. In building these DA status indices for SADs, the expert physician’s global assessment is commonly used as the external criterion and then considered the ‘gold standard’. Thus, to have face validity, these DA indices should have a close correlation with this external standard.

Moreover, since spontaneous or therapy-induced reversibility is considered a peculiarity of DA, the composite indices built to assess it should demonstrate their sensitivity to any change of DA in the clinical course, or at least to be able to appreciate a relevant difference in this disease status entity [22].

DA criteria for SSc were first proposed and validated in multicentre study by the European Scleroderma Study Group (EScSG) in the first years of the 2000s [7, 8]. This EScSG DA index was largely used in following clinical and therapeutic studies, although some aspects of it were criticised. Namely, the face validity of some items has been questioned [23], and no validation of this index was made in an independent cohort. Moreover, no study was carried out to verify the sensitivity to change of this DA scale.

Starting from these considerations, a couple of years ago, the EUSTAR task force derived a new DA index for SSc [10] from the analysis of a cohort of paper patients, extracted from its database. This new EUSTAR DA index was then validated in a second independent cohort of SSc patients, and its sensitivity to change evaluated comparing the overtime variations of this index to those of the Medsger Severity Score [24], which is considered a sensitive index of the severity in this disorder.

In a previous study, we demonstrated that the summed number of MHEs and MTs in NVC (the so-called NEMO score) was a good indicator of steady state DA in SSc, since it was closely correlated with the contemporarily assessed EScSG index [6]. A validation of the NEMO score was made in a subsequent study performed in two independent cohorts of patients [9].

In the present study, the validity of the NEMO score as a good tool to measure steady state DA in patients with SSc has been confirmed by its significant correlation with both the EScSG and EUSTAR composite indices at different times of observation. Moreover, since the differences of the NEMO score between two following observation times were closely correlated with analogous variations obtained by computing at the same times both EScSG and EUSTAR index scores, the NEMO score has also been shown to be capable of capturing the overtime variations of DA and then to be sensitive to the changes of this disease status entity.

To have the possibility of evaluating the activity levels of DA using a relatively simple, non-invasive method as the computation of the number of MHEs and MTs in NVC may offer some advantages in terms of feasibility with respect to the assessment of the same disease status entity which one can obtain by calculating more complex composite index scores. Although the application of both the EScSG and EUSTAR indices is undoubtedly more useful for a more complete evaluation of DA level, since these indices allow the assessment of a large spectrum of clinical and biological features of the disease, the computation of an elevated NEMO score may represent a reliable warning signal of current microvascular derangement, which would alert the physician to perform a more careful clinical work out and take the proper therapeutic measures.

SSc is defined as a disease of the microvascular bed of the skin compartment, which may also involve the small vessels of internal organs. Endothelial damage is considered the first step of the pathological process in SSc, while migration of mononuclear inflammatory cells in the interstitium and the following activation of myofibroblast and fibroblast lineages, with the consequent fibrotic evolution of the compromised tissues and organs, are considered the subsequent phases of the disorder [25–27].

The typical response to the initial microvascular damage in SSc is a loss of capillaries with the compensatory dilatation of surviving capillary loops and formation of enlarged capillaries and GCs in a tentative regenerative process [28]. During the progression of the disorder, the destiny of many capillaries is thrombotic obliteration followed by extravasation. Therefore, multiple MTs and MHEs can be observed in the NVC, aligned distally in the cuticle, in the active phases of the disease, as the result of a synchronous pathological aggression of many capillaries [29]. Thus, it is not surprising that this NVC feature, which can be precisely assessed by computing the NEMO score, can be a strong indicator of active phases of the disease.

Since it has been shown that iloprost infusion may cause some modifications of the microvascular bed, by increasing the number of capillaries visible in NVC [30], the fact that a certain number of patients included in this study were performing this kind of therapy could have partially influenced the results of this work. However, the NEMO score is based only on the computation of MHEs and MTs, and then, this study limitation may be very marginal since there is no demonstration that iloprost may cause changes of these NVC abnormalities.

## Conclusions

The NEMO score may be confirmed as a feasible, non-invasive, and valid tool to assess steady state levels of DA in patients with SSc. Furthermore, the NEMO score has also demonstrated to be sensitive to capture overtime changes of this disease status entity. Thus, the NEMO score could be either an alternative or a complementary method to the previously proposed and validated composite indices for the evaluation DA in SSc patients.

## Supplementary information

**Additional file1: Table S1.** Range of Δ values (T1 minus T0 values) of NEMO score, EScSG and EUSTAR indices subdivided in quartiles. These range values may be indicative of stable DA or overtime changes of this status entity by applying the three different methods of DA evaluation.

## Data Availability

The datasets used and analyses made during the current study are available from the corresponding author on reasonable request.

## Abbreviations

ACA: Anti-centromere antibodies
ANA: Antinuclear antibodies
ACR/EULAR: American College of Rheumatology/EUropean League Against Rheumatism
AUC: Area under the curve
CI: Confidence interval
CRP: C-reactive protein
DA: Disease activity
dcSSc: Diffuse cutaneous systemic sclerosis
DLCO: Diffusing capacity of the lung for carbon monoxide
EScSG: European Scleroderma Study Group
ESR: Erythrocyte sedimentation rate
EUSTAR: European Scleroderma Trial and Research
GCs: Giant capillaries
lcSSc: Limited cutaneous systemic sclerosis
MHEs: Microhaemorrhages
mRSS: Modified Rodnan skin score
MTs: Microthrombosis
NEMO: Number of microhaemorrhages and microthromboses
NVC: Nailfold videocapillaroscopy
ROC: Receiver operating characteristic
SSc: Systemic sclerosis
