# Supplementary material for: NEMO score in nailfold videocapillaroscopy is a good tool to assess both steady state levels and overtime changes of disease activity in patients with systemic sclerosis: a comparison with the proposed composite indices for this disease status entity
Source: Arthritis Res Ther. 2019 Nov 29;21:258. doi: 10.1186/s13075-019-2032-6 (PMC6884889; doi:10.1186/s13075-019-2032-6)
Supplement: Supplementary file 1 — Additional file1: Table S1. Range of Δ values (T1 minus T0 values) of NEMO score, EScSG and EUSTAR indices subdivided in quartiles. These range values may be indicative of stable DA or overtime changes of this status entity by applying the three different methods of DA evaluation. [file 13075_2019_2032_MOESM1_ESM.docx]

**Table S1**

Range of Δ values (T1 minus T0 values) of NEMO score, EScSG and EUSTAR indices subdivided in quartiles. These range values may be indicative of stable DA or overtime changes of this status entity by applying the three different methods of DA evaluation.

|  |  | **Increased DA** | **Stable DA** | **Moderately decreased DA** | **Strongly decreased DA** |
| --- | --- | --- | --- | --- | --- |
|  | **Quartiles** | 1^st^ quartile | 2^nd^ quartile | 3^rd^ quartile | 4^rth^ quartile |
| **NEMO score** | range  (median) | from 30 to 2  (5.5) | from 1 to -1  (0.0) | from -2 to -6  (-4.0) | from -7 to -30  (-12.5) |
| **EScSG index** | range  (median) | from 6.5 to 1  (2.0) | from 0.5 to -0.5  (0.0) | from -1 to -2.5  (-2.0) | from -3 to -8  (4.0) |
| **EUSTAR index** | range  (median) | from 3.1 to 0.6  (1.24) | from 0.5 to -0.5  (0.0) | from -0.6 to -1.9  (-1.5) | from -2 to -5  (2.5) |
